# Dipeptidyl peptidase-4 inhibitor protects against non-alcoholic steatohepatitis in mice by targeting TRAIL receptor-mediated lipoapoptosis via modulating hepatic dipeptidyl peptidase-4 expression

**DOI:** 10.1038/s41598-020-75288-y

**Published:** 2020-11-10

**Authors:** Minyoung Lee, Eugene Shin, Jaehyun Bae, Yongin Cho, Ji-Yeon Lee, Yong-ho Lee, Byung-Wan Lee, Eun Seok Kang, Bong-Soo Cha

**Affiliations:** 1 Division of Endocrinology and Metabolism, Department of Internal Medicine, Yonsei University College of Medicine, Seoul, Republic of Korea; 2 Institute of Endocrine Research, Yonsei University College of Medicine, Seoul, Republic of Korea; 3 Department of Internal Medicine, Inha University School of Medicine, Incheon, Republic of Korea

**Keywords:** Endocrinology, Gastroenterology, Medical research

## Abstract

Dipeptidyl peptidase-4 inhibitors (DPP4i) are antidiabetic medications that prevent cleavage of incretin hormones by dipeptidyl peptidase-4 (DPP4). DPP4 is ubiquitously expressed, and its hepatic DPP4 expression is upregulated under non-alcoholic steatohepatitis (NASH) conditions. We investigated the effect of DPP4i treatment on NASH pathogenesis, as well as its potential underlying molecular mechanisms. Mice were randomly divided into three groups: Group 1, chow-fed mice treated with vehicle for 20 weeks; Group 2, high-fat, high-fructose, and high-cholesterol Amylin liver NASH (AMLN) diet-fed mice treated with vehicle for 20 weeks; Group 3, AMLN diet-fed mice treated with vehicle for the first 10 weeks, followed by the DPP4i teneligliptin (20 mg/kg/day) for additional 10 weeks. DPP4i administration reduced serum liver enzyme and hepatic triglyceride levels and markedly improved hepatic steatosis and fibrosis in the AMLN diet-induced NASH model. In vivo, NASH alleviation significantly correlated with the suppression of tumor necrosis factor-related apoptosis-inducing ligand (TRAIL) receptor-mediated apoptosis and downregulated hepatic DPP4 expression. In vitro, DPP4i treatment significantly decreased the markers of TRAIL receptor-mediated lipoapoptosis and suppressed DPP4 expression in palmitate-treated hepatocytes. In conclusion, DPP4i may efficiently attenuate the pathogenesis of AMLN diet-induced NASH in mice by suppressing lipotoxicity-induced apoptosis, possibly by modulating hepatic DPP4 expression.

## Introduction

Non-alcoholic fatty liver disease (NAFLD) has emerged as a metabolic liver disorder affecting approximately one quarter of the world’s population^1^. NAFLD encompasses a wide range of conditions, from simple steatosis to non-alcoholic steatohepatitis (NASH), the more aggressive form characterized by hepatic steatosis and inflammation due to the hepatocyte injury^2–4^. NASH prevalence varies from 1.5 to 6.45% worldwide, and it is currently the second leading cause of cirrhosis among the adult population awaiting liver transplantation in the US^1,5^. Thus, the development of effective therapeutic strategy to treat NASH is of great clinical interest. Several clinical trials have been recently conducted to evaluate pharmacotherapies for NASH; however, no medications have been approved to date by the US Food and Drug Administration (FDA)^6^.

Dipeptidyl peptidase-4 inhibitors (DPP4i) are widely used oral antidiabetic medications that inhibit the degradation of incretin hormones including glucagon like peptide-1 (GLP-1). Treatment with these agents subsequently increases the glucose-dependent secretion of insulin, inhibits glucagon secretion, and slows down gastric emptying^7^. As dipeptidyl peptidase-4 (DPP4) is ubiquitously expressed cell surface peptidase, modulation of its function may regulate not only glucose metabolism but may also have diverse systemic effects^8^. Liver expresses and secretes DPP4, and the hepatic DPP4 expression levels are associated with a degree of hepatic steatosis in patients with NASH^9,10^. Furthermore, in animal studies, an increase in the hepatic DPP4 expression level promoted NAFLD development^11,12^. Recently, several studies have reported regarding the therapeutic potential of DPP4i to treat NASH in animals and humans^13,14^. However, the molecular mechanisms underlying the effects of DPP4i in NASH alleviation have not been elucidated. In particular, the role of hepatic DPP4 expression during the DPP4i-mediated NASH amelioration has not been concluded yet.

Lipotoxicity due to the elevated levels of cytotoxic saturated free fatty acids (FFAs) such as palmitate causes death of hepatocytes, a process termed as lipoapoptosis^15^. Lipoapoptosis is a pathological hallmark of NASH, and it correlates with the disease severity^15,16^. Increased endoplasmic reticulum (ER) stress caused by these cytotoxic lipids activates death receptors on the cell surface of hepatocytes^16^. Among the death receptors, tumor necrosis factor (TNF)-related apoptosis-inducing ligand receptor 2 (TRAIL-R2, also known as death receptor 5 [DR5]) appears to be a major mediator of lipotoxicity in hepatocytes^15^, inducing caspase-dependent hepatocyte apoptosis^17^. Increased hepatic TRAIL-R2 expression, driven by lipotoxicity-stimulated ER stress, is mediated by the transcription factor CAAT/enhancer binding protein (C/EBP) homologous protein (CHOP)^16^, one of direct target genes of activating transcription factor 4 (ATF4)^18–20^. Interestingly, the ATF4-consensus site lies in the exon 1 of DPP4 gene^21^, suggesting a possible mechanistic link between hepatocyte DPP4 expression and the ER stress-induced lipoapoptosis in the liver. Furthermore, treatment with teneligliptin, a DPP4i, significantly reduced high glucose-induced expression of ER stress markers, including ATF4 and CHOP in endothelial cells in vitro^22^. However, the effect of DPP4i treatment on ER stress-mediated lipoapoptosis in NASH has never been investigated.

In the present study, we used high-fat, high-fructose, and high-cholesterol Amylin liver NASH (AMLN) diet to induce NASH in mice^23^ and palmitate-stimulated hepatocytes. We investigated whether DPP4i could effectively alleviate the pathogenesis of NASH by regulating hepatic DPP4 expression and lipotoxicity-induced apoptosis using in vivo and in vitro models.

## Results

### DPP4i improves biochemical indices and reduces liver fibrosis and pro-inflammatory marker expression in the AMLN diet-induced mouse model of NASH

**Figure 1:**
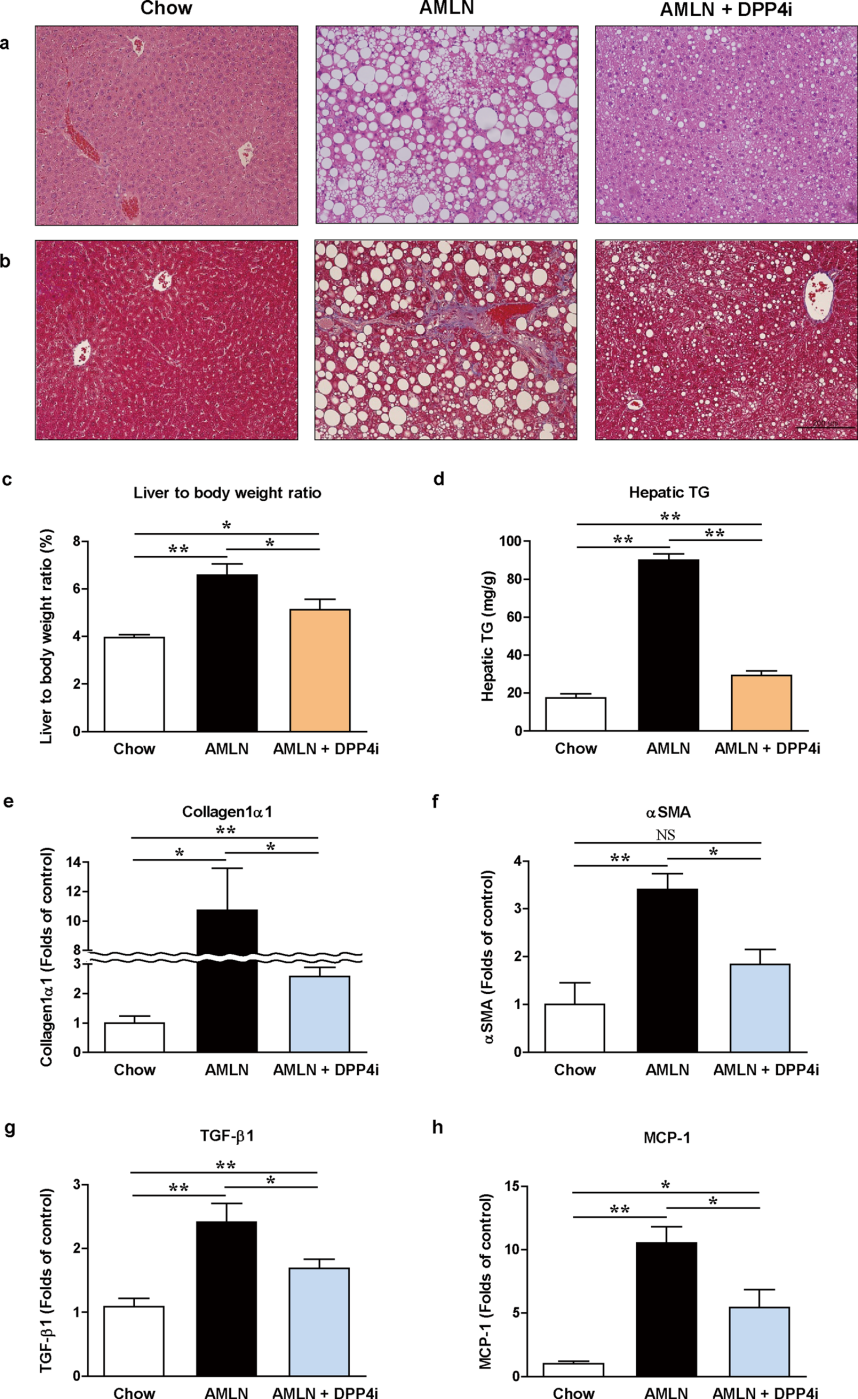
DPP4i ameliorates histopathological phenotype of NASH and reduces fibrotic and pro-inflammatory markers in the AMLN diet-induced NASH mouse model. Representative histopathological images of H&E staining (**a**) and MT staining (**b**) in vehicle-treated chow-fed mice, vehicle-treated AMLN-fed mice, and DPP4i-treated AMLN-fed mice (200 × magnification, scale bar: 200 μm). Graphs showing liver to body weight ratio (**c**), hepatic triglycerides level (**d**), and relative mRNA expression levels of collagen1α1 (**e**), αSMA (**f**), TGF-β1 (**g**), and MCP-1 (**h**) by qPCR according to the diet type and DPP4i administration at 20th week. Data in all graphs are presented as mean ± SEM. *p < 0.05; **p < 0.01; *NS*, not statistically significant. Chow, vehicle-treated chow-fed mice; AMLN, vehicle-treated AMLN-fed mice; AMLN + DPP4i, DPP4i-treated (teneligliptin 20 mg/kg of body weight/day by oral gavage for 10 weeks) AMLN-fed mice.

We evaluated the characteristic features such as body weight and biochemical indices, in response to the AMLN diet and DPP4i administration in male C57BL/6J mice at 20th week (Table 1). Vehicle-treated AMLN-fed mice exhibited a significant increase in body weight and liver to body weight ratio compared with the chow-fed mice (Table 1 and Fig. 1c). The levels of serum total cholesterol, FFAs, aspartate aminotransferase (AST), and alanine aminotransferase (ALT) were also significantly higher in the vehicle-treated AMLN-fed mice than in the chow-fed mice (all *p* < 0.05). Administration of DPP4i significantly reduced the body weight, liver to body weight ratio, and the levels of serum total cholesterol, FFAs, AST, and ALT in the AMLN-fed mice (all *p* < 0.05). Daily food intake, body weight, and random glucose values during the all experimental course are summarized in Supplementary Figure S1b–d.

**Table 1:** Characteristic features of body weight and biochemical indices according to the AMLN diet and DPP4i administration at 20th week.

	Chow	AMLN	AMLN + DPP4i
	N = 10	N = 13	N = 10
Body weight (g)	28.1 ± 1.4	39.4 ± 2.1*	35.1 ± 2.9*^#^
Fasting serum glucose (mg/dL)	156 ± 32	165 ± 36	172 ± 34
Fasting serum insulin (ng/dL)	0.33 ± 0.27	0.25 ± 0.06	0.24 ± 0.04
Serum total cholesterol (mg/dL)	72 ± 7	176 ± 59*	127 ± 40*^#^
Serum free fatty acids (mM)	0.18 ± 0.04	0.65 ± 0.17*	0.43 ± 0.13*^#^
Serum triglyceride (mg/dL)	89 ± 29	38 ± 17*	25 ± 6*^#^
Serum AST (IU/L)	86 ± 47	287 ± 162*	161 ± 60*^#^
Serum ALT (IU/L)	23 ± 4	347 ± 292*	142 ± 121*^#^

Data are presented as mean ± SEM. *p < 0.05 versus vehicle-treated chow-fed mice. ^#^*p* < 0.05 versus vehicle-treated AMLN-fed mice. Chow, vehicle-treated chow-fed mice; AMLN, vehicle-treated AMLN-fed mice; AMLN + DPP4i, DPP4i-treated (teneligliptin 20 mg/kg of body weight/day by oral gavage for 10 weeks) AMLN-fed mice.

ALT, alanine aminotransferase; AMLN, Amylin liver NASH model diet; AST, aspartate aminotransferase; DPP4i, dipeptidyl peptidase-4 inhibitors; NAFLD, non-alcoholic fatty liver disease.

### DPP4i alleviates the pathogenesis of NASH in the AMLN diet-induced mouse model

Histological assessment of hepatic steatosis was performed on the hematoxylin and eosin (H&E)-stained tissue sections, and hepatic fibrosis was evaluated using the Masson’s trichrome (MT)-stained tissue sections (Fig. 1a,b). NAFLD activity score was also assessed using the histological H&E-stained tissue sections (Table 2). H&E staining revealed an increase in steatosis, lobular inflammation, and hepatocyte ballooning in the vehicle-treated AMLN-fed mice compared with the chow-fed mice. Treatment with DPP4i significantly improved the histopathological liver damage and NAFLD activity score in the AMLN-fed mice (Fig. 1a and Table 2). The MT-stained tissue sections revealed that hepatic fibrosis was progressed in the AMLN-fed mice, and DPP4i administration improved fibrosis (Fig. 1b). Increased hepatic triglyceride (TG) levels in the AMLN-fed mice were also significantly reversed by the DPP4i treatment (Fig. 1d,* p*  < 0.01).

**Table 2:** Characteristic features of liver pathology and NAFLD activity score according to the AMLN diet and DPP4i administration at 20th week.

	Chow	AMLN	AMLN + DPP4i
	N = 10	N = 13	N = 10
Steatosis	0.7 ± 0.5	2.8 ± 0.4*	1.9 ± 0.9*^#^
Lobular inflammation	0.1 ± 0.3	1.8 ± 0.6*	1.1 ± 0.7*^#^
Hepatocyte ballooning	0.8 ± 0.4	1.6 ± 0.5*	1.3 ± 0.5*
NAFLD activity score	1.6 ± 0.7	6.2 ± 1.3*	4.3 ± 1.9*^#^

Data are presented as mean ± SEM. *p < 0.05 versus vehicle-treated chow-fed mice. ^#^*p* < 0.05 versus vehicle-treated AMLN-fed mice. Chow, vehicle-treated chow-fed mice; AMLN, vehicle-treated AMLN-fed mice; AMLN + DPP4i, DPP4i-treated (teneligliptin 20 mg/kg of body weight/day by oral gavage for 10 weeks) AMLN-fed mice.

AMLN, Amylin liver NASH model diet; DPP4i, dipeptidyl peptidase-4 inhibitors; NAFLD, non-alcoholic fatty liver disease.

We analyzed the mRNA expression of collagen type 1 α1 (collagen1α1) and α-smooth muscle actin (αSMA), the markers of fibrosis, and transforming growth factor-1 β (TGF-1β) and monocyte chemoattractant protein-1 (MCP-1), the pro-inflammatory markers, in the liver (Fig. 1e–h). The levels of fibrotic and pro-inflammatory markers were significantly higher in the vehicle-treated AMLN-fed mice than in the chow-fed mice (all *p* < 0.05). DPP4i treatment reduced liver fibrosis and the expression levels of pro-inflammatory markers in the AMLN-fed mice (all *p* < 0.05).

### DPP4i downregulates hepatic DPP4 expression and attenuates TRAIL-R2-mediated apoptosis in the AMLN diet-induced mouse model of NASH

**Figure 2:**
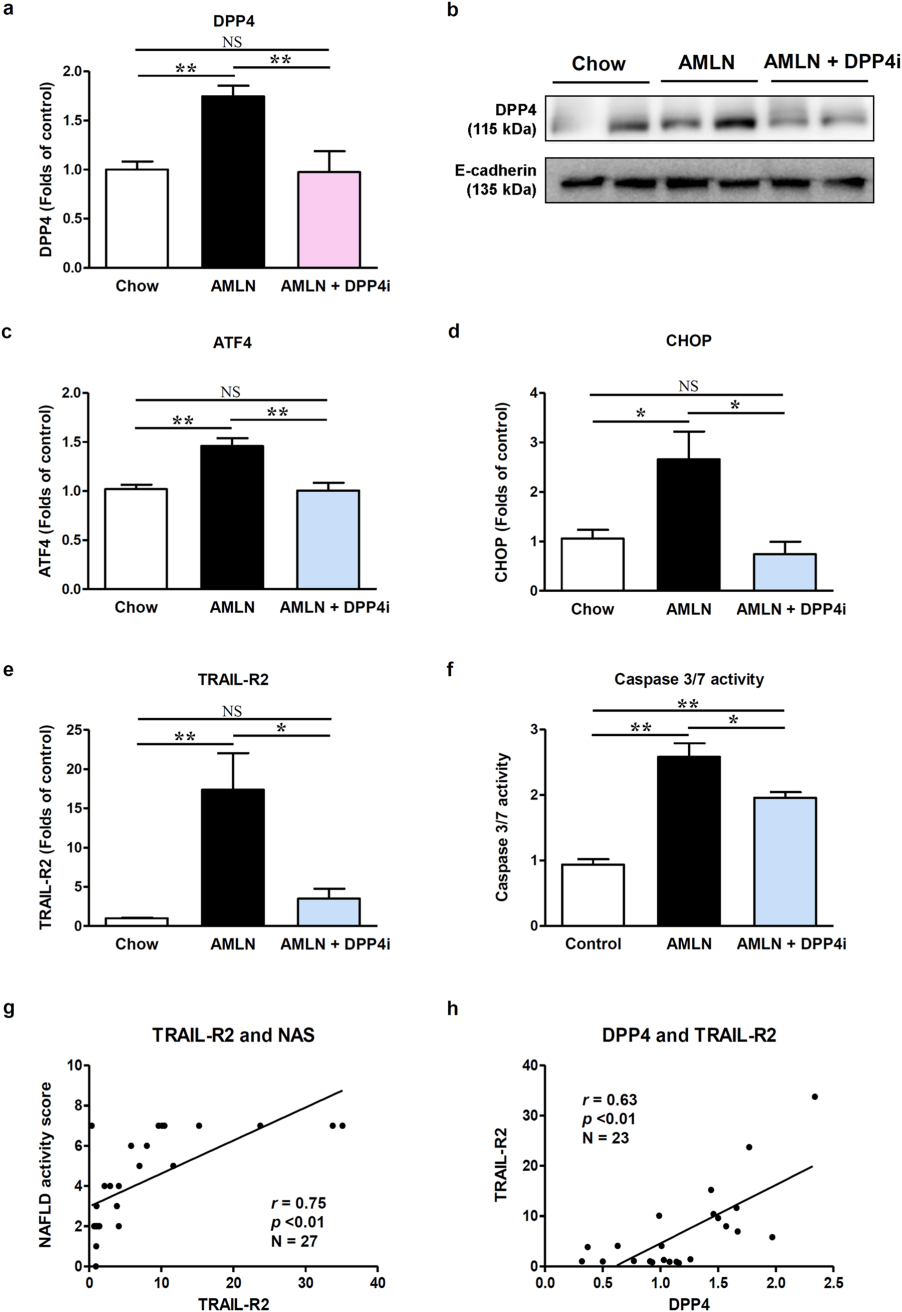
DPP4i decreases hepatic DPP4 expression and attenuates TRAIL-R2-mediated apoptosis in the AMLN diet-induced NASH mouse model. Graphs showing liver DPP4 mRNA levels (**a**) by qPCR and protein level (**b**) by western blot according to the diet type and DPP4i administration. Relevant bands from different western blots were cropped and grouped together. Original blots are shown in Supplementary Figure S5. Graphs showing relative mRNA expression levels of ATF4 (**c**), CHOP (**d**), and TRAIL-R2 (**e**) determined by qPCR according to the diet type and DPP4i administration. A graph showing liver caspase 3/7 activity (**f**) according to the diet type and DPP4i administration. Correlation plots between relative TRAIL-R2 mRNA levels and NAFLD activity score (**g**), and between relative mRNA levels of DPP4 and TRAIL-R2 (**h**). Data in all graphs are presented as mean ± SEM. *p < 0.05; **p < 0.01; NS, not statistically significant. Chow, vehicle-treated chow-fed mice; AMLN, vehicle-treated AMLN-fed mice; AMLN + DPP4i, DPP4i-treated (teneligliptin 20 mg/kg of body weight/day by oral gavage for 10 weeks) AMLN-fed mice; NAS, NAFLD activity score.

We measured the hepatic mRNA and protein levels of DPP4 (Fig. 2a,b), as hepatic DPP4 was previously shown to be elevated during NASH^10,24^. In comparison with the chow-fed mice, vehicle-treated AMLN-fed mice showed upregulated mRNA and protein expression of DPP4 in the liver; however, their expression was downregulated by the DPP4i treatment. As the ATF4-consensus site in the exon 1 of DPP4 gene was previously identified^21^, we analyzed the mRNA expression of ATF4 and its downstream target, CHOP (Fig. 2c,d). Consistent with the DPP4 mRNA expression results, ATF4 and CHOP mRNA expression levels were higher in the vehicle-treated AMLN-fed mice than in the chow-fed mice, and DPP4i treatment attenuated these changes (all *p* < 0.05).

To investigate whether the increase in the ER stress markers (ATF4 and CHOP) causes ER stress-mediated apoptosis, we evaluated TRAIL-R2 mRNA levels and caspase 3/7 activity in the liver tissues (Fig. 2e,f). Vehicle-treated AMLN-fed mice showed a prominent increase in the TRAIL-R2 expression levels and caspase 3/7 activity compared with the chow-fed mice (all *p* < 0.01). Consistent with the effects on ER stress markers, DPP4i attenuated TRAIL-R2 expression and caspase 3/7 activity in the liver of AMLN-fed mice. Interestingly, the relative TRAIL-R2 mRNA expression significantly correlated with NAFLD activity score (Fig. 2g, *p* < 0.01), suggesting that the death receptor signaling via TRAIL-R2 plays a key role in the pathogenesis of AMLN diet-induced NASH. Furthermore, the expression of DPP4 mRNA significantly correlated with TRAIL-R2 expression levels, biochemical indices, and NAFLD activity score (Fig. 2h and Table 3). Even after adjustment for confounding biochemical indices, the relative DPP4 mRNA expression level was a significant determinant factor for a higher NAFLD activity score (Table 4).

**Table 3:** Correlation between the relative DPP4 mRNA expression levels, biochemical indices, and the NAFLD activity score.

N = 29	Liver DPP4 mRNA	
	*r*	*p*-value
Body weight (g)	0.51	< 0.01
Liver weight (g)	0.57	< 0.01
Liver to body weight (%)	0.57	< 0.01
NAFLD activity score	0.55	< 0.01
Hepatic triglyceride (mg/g)	0.64	< 0.01
Fasting serum glucose (mg/dL)	0.16	0.42
Fasting serum insulin (ng/dL)	0.08	0.67
Serum total cholesterol (mg/dL)	0.56	< 0.01
Serum free fatty acids (mM)	0.40	0.05
Serum AST (IU/L)	0.70	< 0.01
Serum ALT (IU/L)	0.63	< 0.01
TRAIL-R2 mRNA	0.63	< 0.01

Data are presented as Spearman’s rank correlation coefficient (*r*). *p* < 0.05 was regarded as statistically significant.

ALT, alanine aminotransferase; AST, aspartate aminotransferase; DPP4, dipeptidyl peptidase-4; NAFLD, non-alcoholic fatty liver disease; TRAIL-R2, TNF-related apoptosis-inducing ligand receptor 2.

**Table 4:** Relative DPP4 mRNA expression as a determinant factor for a higher NAFLD activity score.

	NAFLD activity score R^2^ = 0.856		
	Regression coefficient	SE	*p*-values
Body weight (g)	**0.302**	**0.056**	** < 0.001**
Serum fasting glucose (mg/dL)	− 0.009	0.007	0.888
Serum AST (IU/L)	− 0.002	0.006	0.708
Serum ALT (IU/L)	< 0.001	0.003	0.946
DPP4 mRNA expression	**1.903**	**0.557**	**0.003**

Multiple linear regression analysis was performed. Bold represent statistically significant values (*p* < 0.05).

ALT, alanine aminotransferase; AST, aspartate aminotransferase; DPP4, dipeptidyl peptidase-4; NAFLD, non-alcoholic fatty liver disease.

We performed TUNEL assay to count apoptotic cell number in the livers of AMLN-fed and chow-fed mice (Supplementary Figure S2). The vehicle-treated AMLN-fed mice showed a six-fold greater increase in the number of TUNEL-positive cells in the liver than the chow-fed mice, and DPP4i treatment significantly reduced that number (Supplementary Figure S2b, *p* < 0.01).

### DPP4i treatment exerts protective effects against lipoapoptosis in hepatocyte

**Figure 3:**
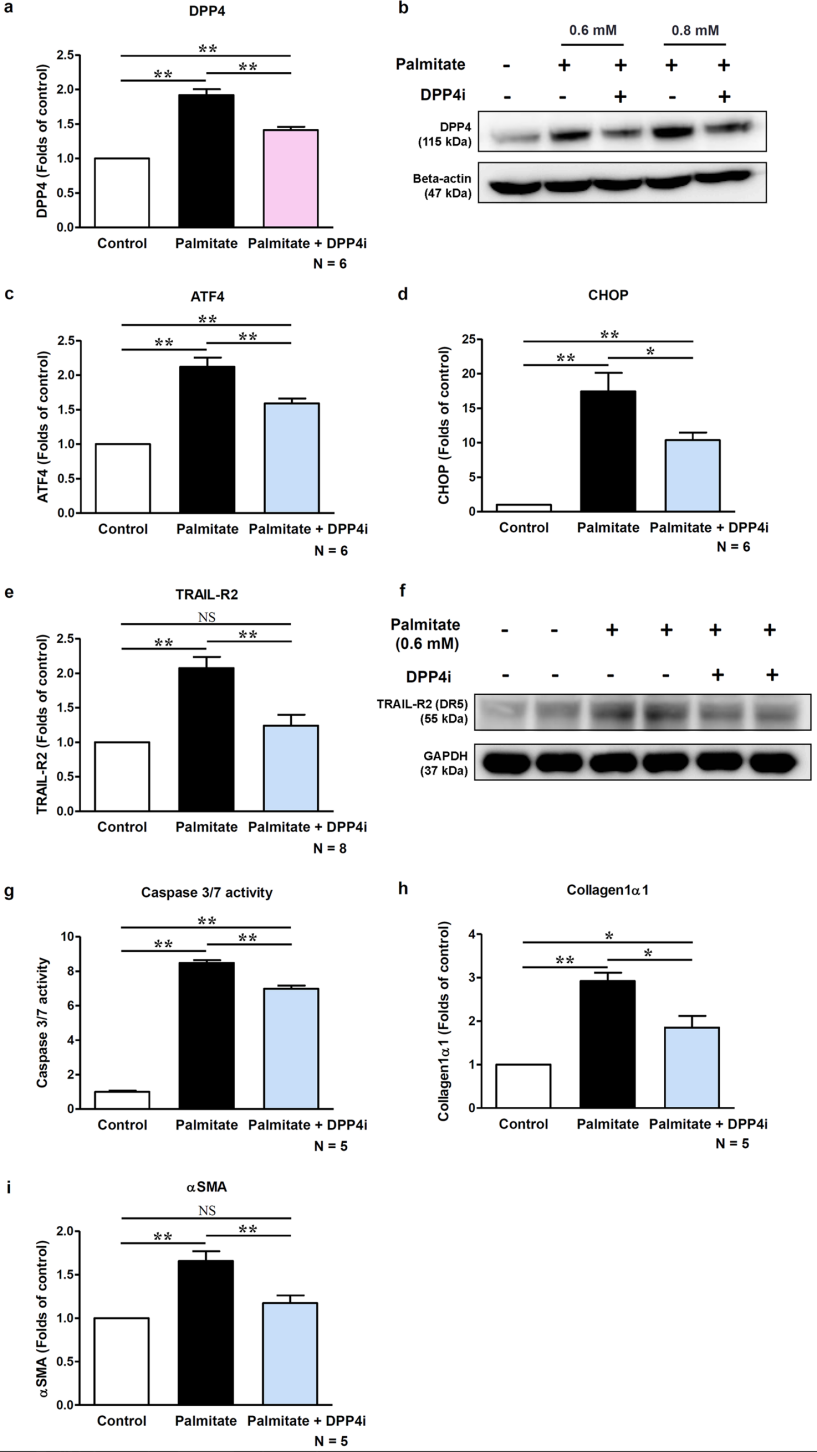
DPP4i treatment protects against lipoapoptosis in hepatocyte. Graphs showing relative mRNA levels of (**a**) DPP4 by qPCR (n = 6) and (**b**) protein level of DPP4 by western blot according to palmitate and DPP4i treatment. Relevant bands from different blots were cropped and grouped together. Original blots are shown in Supplementary Figure S6. Graphs showing relative mRNA levels of ER stress markers (**c**) ATF4, (**d**) CHOP (n = 6 per each marker), and (**e**) TRAIL-R2 (n = 8) by PCR, and (**f**) protein level of TRAIL-R2 (death receptor 5, DR5) by western blot according to palmitate and DPP4i treatment. Relevant bands from different blots were cropped and grouped together. Original blots are shown in Supplementary Figure S7. Graphs showing (**g**) caspase 3/7 activity (n = 5), and relative mRNA expression levels of (**h**) collagen1α1 (n = 5), and (**i**) αSMA (n = 5) by qPCR according to palmitate and DPP4i treatment. Data in all graphs are presented as mean ± SEM. *p < 0.05; **p < 0.01; *NS* not statistically significant. Control, HepG2 cells, no treatment; Palmitate, HepG2 cells treated with palmitate (0.6 mM) for 18 h; Palmitate + DPP4i, HepG2 cells treated with teneligliptin (3 μM) for 6 h, followed by palmitate treatment (0.6 mM) for 18 h. Each experiment was repeated as a described number of times in the figure.

Next, we evaluated the effect of DPP4i treatment on DPP4 expression, ER stress markers, and TRAIL-R2-mediated apoptosis using HepG2 cells (Fig. 3). Palmitate-treated hepatocytes showed a significant increase in DPP4, ATF4, and CHOP mRNA expression levels, as well as DPP4 protein expression level (Fig. 3a–d). Treatment with DPP4i significantly attenuated the increased mRNA and protein levels of DPP4 as well as the mRNA levels of ATF4 and CHOP in the palmitate-treated hepatocytes. Palmitate treatment also increased the mRNA and protein levels of TRAIL-R2 and effector caspase 3/7 activity in hepatocytes, thereby promoting apoptosis via TRAIL-R2 (Fig. 3e–g). TRAIL-R2 expression and caspase 3/7 activity, the hallmarks of TRAIL-R2-mediated apoptosis, were markedly suppressed by the DPP4i treatment. The mRNA expressions of collagen1α1 and αSMA were also significantly increased by palmitate treatment (Fig. 3h,i, all *p* < 0.01), and this effect was significantly reduced by DPP4i (all *p* < 0.05). To evaluate whether DPP4i suppresses TRAIL-R2-mediated lipoapoptosis in general or palmitate-activated specific conditions, we cultured hepatocytes with recombinant human TRAIL protein (Supplementary Figure S3). The addition of TRAIL markedly increased caspase 3/7 activity in a dose dependent manner, and the DPP4i treatment significantly attenuated these changes (Supplementary Figure S3a). Similar to caspase 3/7 activity results, cell viability analyzed using the CCK8 assay showed a gradually decreasing trend in a TRAIL concentration-dependent manner. Administration of DPP4i significantly improved cell viability at high TRAIL concentration (Supplemental Figure S3b).

Taken together, these results indicate that DPP4i treatment may suppress DPP4 expression, as well as TRAIL-R2-mediated lipoapoptosis, in hepatocytes, suggesting that DPP4 expression is associated with TRAIL-R2-mediated lipoapoptosis.

### DPP4i treatment is associated with improvement in brown adipose tissue function and decreased visceral adipose tissue inflammation

The extrahepatic effect of DPP4i on adipose tissue in vivo was also evaluated to explain the preventive effect of DPP4i on body weight gain^25,26^. To examine brown adipose tissue (BAT) function, BAT weights and mRNA expression of BAT thermogenic markers including uncoupling protein-1 (UCP-1), PPAR-γ coactivator 1-alpha (PGC1α), and transcriptional regulator PR domain containing 16 (PRDM16), were evaluated (Supplementary Figure S4a,b)^25^. UCP-1 expression was significantly downregulated in the vehicle-treated AMLN-fed mice compared with the chow-fed mice (*p* = 0.018). The expression levels of PGC1α and PRDM16 were decreased in the vehicle-treated AMLN-fed mice compared with the chow-fed mice without statistical significance. DPP4i treatment significantly increased the expression levels of UCP-1 and PGC1α in the AMLN-fed mice. We also evaluated the mRNA expression of adipose tissue inflammatory markers such as interleukin (IL)-6, MCP-1, and TNF-α using the perirenal visceral adipose tissue (VAT; Supplementary Figure S4c)^21^. The levels of all these markers were significantly elevated in the vehicle-treated AMLN-fed mice compared with the chow-fed mice. DPP4i administration significantly decreased IL-6 level in the AMLN-fed mice (*p* = 0.015). MCP1 and TNF-α levels were also decreased by DPP4i in the AMLN-fed mice, even though statistical significance was not achieved. Collectively, DPP4i treatment partially contributed to the improvement of BAT function and decreased VAT inflammation in the present study, potentially providing an explanation for the attenuated body weight gain in the AMLN-fed mice.

## Discussion

There is a clinical need to develop a potential therapeutic candidate to treat NASH. In the present study, we demonstrated that the administration of DPP4i ameliorated the pathogenesis of NASH in the AMLN diet-induced mouse model, suppressed lipoapoptosis, and downregulated hepatic DPP4 expression.

DPP4i are useful antidiabetic medications considered to exert pleiotropic metabolic effects including amelioration of NASH^8^. The potential nonglycemic actions of DPP4i in diverse biological physiologies may be attributed to the ubiquitous expression of DPP4 in various organs^27^. DPP4 is a 110-kDa membrane-associated peptidase that also exists in a slightly smaller, soluble form^28^. DPP4 is expressed in all organs including the small intestine, biliary tract, and exocrine pancreas, while the liver is one of the organs with high expression of DPP4^27,29,30^. In previous reports, hepatic DPP4 expression was significantly higher in patients with NAFLD than in the healthy controls^24^, and correlated with the histopathologic grade of NASH^10^. These results indicate that hepatic DPP4 expression may increase in response to metabolically toxic stimuli, thereby contributing to NASH development. In the present study, we confirmed the upregulation of hepatic DPP4 expression both in vivo (in the AMLN diet-induced NASH mouse model) and in vitro (in palmitate-stimulated HepG2 cells).

The increase in hepatic DPP4 expression may contribute to liver injury during NASH development in several ways. Previous reports have mainly focused on the increased lipogenesis in the liver associated with upregulated DPP4 expression^27,31^. Mice and rats lacking DPP4 activity exhibited decreased levels of hepatic sterol regulatory element-binding transcription factor 1-c (SREBP-1c) and fatty acid synthase (FAS) expression^32,33^. The administration of DPP4i inhibited hepatic lipogenic gene expression and enhanced AMP-activated protein kinase (AMPK) activity in ob/ob and diet-induced NAFLD mouse models^14,34,35^. Other researchers reported that DPP4i downregulated leukocyte cell-derived chemotaxin 2 (LECT2) expression in the livers of high fat diet-fed mice^36^. As LECT2 induces SREBP-1c cleavage, and lipid accumulation, DPP4i-treated mice showed improved hepatic steatosis. However, modulation of lipogenesis is not sufficient to explain the whole mechanisms underlying the protective effects of DPP4i against NASH, as the development of steatosis is a benign and reversible state and requires triggering of different inflammatory signals to transform into NASH^37^. The activation of inflammation-mediated cells such as Kupffer cells and hepatic stellate cells (HSCs) plays an important role in the transition from steatosis and NASH via the pro-inflammatory and pro-fibrotic factors, including TNFα, TGF-β, collagen1α1, and αSMA^38,39^. In the present study, we sacrificed one mouse each from the control and the AMLN-fed groups at 10th weeks of diet administration to assess liver histopathology before proceeding with the DPP4i administration and found that 10 weeks of AMLN diet maintainance had already induced hepatic steatosis and inflammation (Supplementary Figure S1e). Thus, our results indicate that the beneficial effects of DPP4i on NASH may be attributed not only to the suppression of hepatic lipogenesis before the development of hepatic steatosis but also to the protection against the progression of hepatic inflammation and fibrosis, even when the early pathological changes of NASH have already been induced. We also demonstrated the DPP4i-mediated reduction of pro-inflammatory and pro-fibrotic factors in vivo and in vitro, indicating that DPP4i modified NASH progression beyond the regulation of steatosis.

Increased DPP4 expression levels in hepatocytes could be responsible for promoting intracellular ER stress through several pathways and DPP4i might suppress these pathways. Overexpression of hepatic DPP4 upregulated lipogenic gene expression with lipid accumulation, resulting in increased intracellular ER stress^11,40,41^. Furthermore, the hepatic DPP4 overexpression leads to further increase of intracellular ER stress by inhibiting the hepatocyte nuclear factor 1b (HNF1b)-associated suppression of superoxide generation^40^. Similar to our findings, DPP4i significantly alleviated ER stress indicators in both high fat diet- and methionine/choline-deficient diet-induced NASH mouse models in previous studies^42,43^.

In addition to hepatocytes, the protective effect of DPP4i against NASH might also be mediated by the modulation of HSCs. Liver fibrosis of NASH is characterized by the excessive accumulation of extracellular matrix, as well as activated HSCs expressing DPP4 on its surface^44,45^. Interestingly, the DPP4 expression was only observed in the activated, but not in the quiescent HSCs, indicating that it might play a role in HSC activation^45^. Indeed, the use of DPP4i markedly inhibited liver fibrosis development via the suppression of HSC proliferation and collagen synthesis^45^. Thus, DPP4i treatment-induced improvement of NASH and decreased hepatic DPP4 expression in our in vivo model might be driven not only from the effect on the hepatocytes but also from that on the HSCs.

To the best of our knowledge, this is the first study to investigate the protective effects of DPP4i on NASH in the context of TRAIL-R2-mediated lipoapoptosis. Hepatic lipotoxicity is a cardinal feature of NASH^17^. Hepatocyte injury caused by FFAs contributes to hepatocyte apoptosis, which can also be referred as lipoapoptosis^46^. Hepatocyte apoptosis is the most common and best-characterized cell death pathway in NASH^17^ and is considered to be the cellular mechanism that distinguishes NASH from steatosis^17,46,47^. The magnitude of hepatocyte apoptosis correlates with the severity of inflammation and fibrosis in NASH^47^. Death receptor signaling via TRAIL-R2 has emerged as a key mechanism underlying hepatocyte lipoapoptosis in NASH^17^. TRAIL-R2-mediated hepatocyte apoptosis is closely related to persistent ER stress induced by FFAs^17,19^. Under sustained ER stress, the expression of ATF4 is upregulated, leading to CHOP transcription^48^, and subsequent increased expression of TRAIL-R2, one of the important downstream target genes of CHOP^17,19^. Enhanced TRAIL-R2-mediated apoptotic signaling during ER stress contributes to pro-inflammatory responses by various chemokines^15^. DPP4 may be involved in the TRAIL-R2-mediated apoptotic cascades in relation to ER stress, considering the association between DPP4 and intracellular ER stress. Meanwhile, based on our in vitro results obtained from the TRAIL-treated hepatocytes, it is also possible that there is a direct interaction between DPP4 and the TRAIL signaling pathways, and DPP4i treatment suppresses this interaction. As the activation of TRAIL signaling pathway causes mitochondrial dysfunction, intracellular reactive oxygen species (ROS) generation, and increased ER stress in return^16,47^, the inhibition of TRAIL signaling pathway by modulating its interaction with DPP4 may result in the downregulation of this vicious cycle. To elucidate a pathophysiological association between DPP4 and the TRAIL signaling pathway, further studies should be conducted.

Although DPP4 inhibitors were developed to suppress the plasma activity of DPP4, they may also downregulate the expression of DPP4 in other organs. For example, in the kidney of diabetic mice, the membrane-bound DPP4 and the cell surface protein integrin β1 are known to interact to induce the formation of TGF-β receptor heterodimer, thereby promoting kidney fibrosis^49^. Treatment with DPP4i inhibited interaction between membrane-bound DPP4 and integrin β1 and decreased renal DPP4 expression levels in the diabetic kidney mouse model, possibly by suppressing the endothelial-to-mesenchymal transition pathway loop^49^. In the present study, the increased levels of hepatic DPP4 might have induced the ER stress-driven TRAIL signaling pathway^11,40,41^ and the enhanced TRAIL-R-induced apoptotic pathway could have further augmented ER stress by upregulating ATF4^16,47^. As a result, DPP4 expression would have been subsequently increased in a positive feedback loop of ER stress, in response to the possible transcriptional upregulation by ATF4^21^. DPP4i, in turn, may block this feedback loop, alleviating both TRAIL-R-induced apoptosis and DPP4 expression in NASH. Similar downregulation of hepatic DPP4 expression by DPP4i was also reported in another study with using NASH animal model^43^.

The AMLN diet used in the present study has higher proportions of fructose and trans-fat compared with the conventional high-fat diet. As a result, the AMLN diet induces NASH development more effectively, along with augmented ER stress and TRAIL signaling pathway, than the conventional high-fat diet. Fructose metabolism in liver leads to a transient fall in adenosine triphosphate (ATP) levels, which does not occur during glucose metabolism^50^. This decrease in ATP levels induces oxidative stress, mitochondrial dysfunction, and uric acid generation, resulting in increased lipid accumulation, ER stress, and NAFLD development^50^. A deletion of TRAIL prevented NAFLD development in mice fed with high-fructose diet, indicating that the high fructose-induced hepatic steatosis and inflammation might be largely mediated by the TRAIL signaling pathway^51^. In addition, current ample of evidence supported that the addition of cholesterol and trans-fatty acids to the diet are critical factors for steatohepatitis progression to fibrosis^52^. Consumption of trans-fats causes proinflammatory cytokine production by monocytes/macrophages, marked hepatic lipid accumulation, and an accretion of lipotoxic metabolites in the liver, reflecting mitochondrial metabolic overload^53–55^. These changes consequently lead to ROS production and oxidative damage followed by the TRAIL-induced apoptosis in hepatocytes^16,56^. Meanwhile, the AMLN diet was reported to have a minimal effect on serum glucose level^23,57^. Hence, the effects of DPP4i on AMLN diet-induced NASH model might be exerted through glucose-independent mechanisms against ER stress and TRAIL-R2-mediated apoptotic pathway.

In the in vivo model used in this study, the extrahepatic effect of DPP4i on adipose tissues might explain the preventative effects of DPP4i on body weight gain, indirectly contributing to the amelioration of NASH. Similar to our results, DPP4i treatment attenuated body weight gain in previous animal studies^25,26^. DPP4 is expressed in adipose tissue, and it is now considered a novel adipokine that may negatively affect insulin senstivity^58,59^. DPP4 expression was positively correlated with the body mass index (BMI) and VAT, and the circulating DPP4 positively correlated with the amount of VAT, adipocyte size, and adipose tissue inflammation^58^. Thus, the inhibition of DPP4 might induce body weight reduction via ameliorating inflammation and insulin resistance in adipocytes. Furthermore, this inhibition of DPP4 may be associated with increased energy expenditure through the activation of BAT function^25^. Soluble DPP4 suppresses β-adrenoreceptor-stimulated UCP-1 expression in adipocytes, and this suppression was prevented by DPP4i treatment^25^. In the present study, DPP4i improved BAT function and VAT inflammation, but the effect was marginally significant. The lesser effect of DPP4i on adipose tissue in the present study compared with that in the previous studies may be due to the difference in the diet type used in animal models. As the proportion of fat in total diet kcal affects weight gain^60^, the AMLN diet might demonstrate a smaller effect on body weight gain and adiposity as a result of its relatively low fat composition (45% kcal fat) compared with the conventional high-fat diet (60% kcal fat) commonly used in the obesity animal models. Thus, in our diet model, the effects of DPP4i on adipose tissue might be diminished compared with the previous reports^25,26^. Although we did not investigate in this study, DPP4i may exert its preventive effects against body weight gain and NAFLD, potentially by playing a role in the gut. DPP4i promoted macrophage-to-feces reverse cholesterol transport and reduced intestinal cholesterol absorption in an obese mice model^61^. DPP4i also exhibited beneficial effects at the intestinal level by modulation of gut microbiota and preservation of ileal crypt depth, thereby decreasing the levels of hepatic proinflammatory cytokines^62^.

We observed significant differences in body weight between vehicle-treated and DPP4i-treated AMLN-fed groups at 7th and 8th weeks; it might be due to the metabolically healthier innate traits of mice in the DPP4i-treated AMLN-fed group than of those in the vehicle-treated AMLN-fed group owing to individual variations within the species. However, there was no significant difference in the body weights between the two groups at 9th week, a week prior to the start of DPP4i administration. Thus, we presumed that the metabolic variations between the two groups would not have been considerably different to affect the current results.

In the current study, we found that DPP4i could effectively ameliorate the pathogenesis of NASH in a mouse model; however, previous human studies failed to show consistent results when treating NASH with DPP4i^13,63,64^. This discrepancy could result from the difference between the species such as mice and human. Another explanation is the dose of teneligliptin used in the current study: it was higher compared with the clinically used human dose (after the drug dose conversion between mice and human is calculated)^65^. Considering our data, to exert beneficial effects on NASH by DPP4i in humans, higher doses than the conventionally used clinical dose of DPP4i might have to be administered. Differences in several important confounding factors such as food intake, BMI, and glucose levels between previous human studies could also contribute to inconsistent results. Future well-designed human clinical trials with sufficient number of subjects and with various doses of DPP4i are necessary to prove their effects on NASH.

In conclusion, we demonstrated that DPP4i alleviated the pathogenesis of AMLN diet-induced NASH in a mouse model through the suppression of lipoapoptosis, possibly by regulating hepatic DPP4 expression. The in-depth mechanism linking hepatic DPP4 and lipoapoptosis needs further investigation to develop an effective therapeutic strategy for NASH.

## Materials and methods

### Animal experiments

Six-week-old male C57BL/6J mice were purchased from the Jackson Laboratory (Bar Harbor, ME, USA). After a 1-week acclimatization period, the mice were randomly assigned to one of three groups depending on the diet and drug treatment: Group 1, chow-fed mice with vehicle treatment (0.5% carboxymethyl cellulose [CMC] solution; Sigma-Aldrich) for 20 weeks (n = 10); Group 2, AMLN-fed mice with vehicle treatment for 20 weeks (n = 13); Group 3, AMLN-fed mice with vehicle treatment for 10 weeks followed by DPP4i (teneligliptin 20 mg/kg of body weight/day by oral gavage) treatment for 10 weeks (n = 10). The experimental protocol is outlined in Supplementary Figure S1a. Regular chow diet (PicoLab Rodent Diet 20 [5053]) contained 23.6% protein, 64.5% carbohydrate, and 11.9% fat (% of total kcal), and the AMNL diet (Catalog number D09100301; Research Diets, New Brunswick, NJ, USA) contained 20% protein, 40% high fat (of these 18% trans-fat), 40% carbohydrates (% of total kcal), and high cholesterol (2% by wt.). Of the total carbohydrate content, fructose constituted 22% by weight. The mice were maintained at a temperature of 23 °C ± 2 °C and humidity level of 60% ± 10% under a 12-h light/dark cycle.

In all cases, animals were anesthetized and sacrificed 24 h after the final administration. After mice were sacrificed, blood was collected via heart puncture and tissues were harvested. Specimens were snap-frozen in liquid nitrogen and maintained at − 80 °C until analysis. All animal experiments were approved by the Institutional Animal Care and Use Committee of Yonsei University Health System (YUHS-IACUC) regulations and guidelines in accordance with the Animal Protection Act (2008), the Laboratory Animal Act (2008), and the Eighth Edition of the Guide for the Care and Use of Laboratory Animals of NRC (2011).

### Cell culture and treatment

The human hepatoma cell line HepG2 (American Type Culture Collection [ATCC], Manassas, VA, USA) was cultured in low glucose Dulbecco’s modified Eagle’s medium (DMEM; SH30021.01, HyClone Laboratories, Logan, UT, USA) supplemented with 10% fetal bovine serum, 1% penicillin, and 1% streptomycin in a 5% CO_2_ incubator at 37 °C. Cells were incubated in a humidified atmosphere at 37 °C and 5% CO_2_ and cultured for 3 days to achieve 70% confluence before treatment with palmitate or DPP4i (teneligliptin). HepG2 cells were treated with palmitate (Sigma-Aldrich, 0.6 mM) for 18 h with or without pre-treatment with DPP4i (teneligliptin, 3 μM) for 6 h and were then analyzed. The mRNA and protein expression levels of DPP4, ER stress, and TRAIL-R2-mediated apoptosis markers were assessed. The mRNA levels of collagen1α1 and αSMA, the typical mesenchymal cell markers corresponding to the activated hepatocyte state that involves the deposition of extracellular matrix during liver fibrosis^66^, were also analyzed. To analyze the effect of DPP4i on TRAIL-mediated apoptosis, HepG2 cells were incubated with the recombinant human TRAIL/TNFSF10 protein (TRAIL; Cat# 375-TL-010, R&D Systems) at concentrations of 50 and 100 ng/mL for 18 h, with or without pre-treatment with DPP4i (teneligliptin, 3 μM) for 6 h.

### Histology and terminal deoxynucleotidyl transferase dUTP nick-end labeling (TUNEL) assay of the liver tissues

For histological assessment of liver sections under a light microscope (Olympus BX40, Olympus Optical Co. Ltd., Tokyo, Japan), 5 mm × 5 mm sections were fixed in 4% paraformaldehyde for 48 h, and embedded in paraffin. Tissue Sections (4 μm) were prepared using a microtome (Reichert Scientific Instruments, Buffalo, NY) and placed on glass slides. H&E staining was performed according to the standard protocols. Liver fibrosis was assessed using the MT staining. The NAFLD activity score and fibrosis stage were evaluated in a blinded manner under 200 × magnification according to the scoring system by Kleiner et al.^67^ The TUNEL assay (In Situ Cell Death Detection Kit, Roche, IN, USA) was performed on the paraffin-embedded liver sections. To quantify the apoptotic cells, the TUNEL-positive nuclei were counted in a total of 40 random microscopic fields (200 × magnification).

## Supplementary information

Supplementary Information.

## Data Availability

All data generated or analyzed during this study are included in this published article.
