# Supplementary material for: Dipeptidyl peptidase-4 inhibitor protects against non-alcoholic steatohepatitis in mice by targeting TRAIL receptor-mediated lipoapoptosis via modulating hepatic dipeptidyl peptidase-4 expression
Source: Sci Rep. 2020 Nov 10;10:19429. doi: 10.1038/s41598-020-75288-y (PMC7655829; doi:10.1038/s41598-020-75288-y)
Supplement: Supplementary file 1 — Supplementary Information. [file 41598_2020_75288_MOESM1_ESM.docx]

**Dipeptidyl peptidase-4 inhibitor protects against non-alcoholic steatohepatitis in mice by targeting TRAIL receptor-mediated lipoapoptosis via modulating hepatic dipeptidyl peptidase-4 expression**

Running title: Dipeptidyl peptidase-4 inhibitors for non-alcoholic steatohepatitis treatment

**Minyoung Lee^1,#^,** **Eugene Shin^2,#^,** Jaehyun Bae^1^, Yongin Cho^3^, Ji-Yeon Lee^1^, Yong-ho Lee^1^, Byung-Wan Lee^1^, Eun Seok Kang^1^, Bong-Soo Cha^1,2,^*

^1^Department of Internal Medicine, Yonsei University College of Medicine, Seoul, Korea

^2^Institute of Endocrine Research, Yonsei University College of Medicine, Seoul, South Korea

^3^Department of Internal Medicine, Inha University College of Medicine, Incheon, Korea.

#Co-first authors

*Corresponding author: Bong-Soo Cha

Division of Endocrinology and Metabolism

Department of Internal Medicine, Yonsei University College of Medicine, 50-1, Yonsei-ro, Seodaemun-gu, Seoul 03722, Republic of Korea

Tel: 82-2-2228-2265, Fax: +82-2-393-6884, E-mail: bscha@yuhs.ac

**Supplemental Appendix**

**Materials and methods**

**Insulin ELISA**

Serum insulin levels were measured using the enzyme-linked immunosorbent assay (ELISA) kit (ALPCO, Inc., Keewaydin Dr, Salem, USA) according to the manufacturer’s instructions.

**Measurement of Caspase 3/7 activity in liver homogenates and cells**

To assess apoptosis in liver homogenates, caspase 3/7 activity was detected using Caspase-Glo 3/7 assay system (#G8091, Promega, Madison, USA) according to the manufacturer’s instructions. Briefly, an equal volume of liver homogenate (5 µg/µL protein) was mixed with the Caspase-Glo Reagent in 96-well, white-walled plates. The reaction mixture was incubated at room temperature for 1.5 h, and the luminescence was measured using a plate-reading luminometer. For cellular caspase-3/-7 activity analysis, cells were seeded in 96-well plates and caspase-3/-7 activation in HepG2 cells was evaluated using the Apo-ONE homogeneous caspase-3/-7 kit (Promega, Madison, WI) according to the manufacturer’s instructions.

**Cell Counting Kit-8 (CCK8) assay**

HepG2 cells were treated with trypsin and seeded into 96-well plates at a density of 3 × 10^3^ cells/well. After 24 h, HepG2 cells were treated with recombinant human TRAIL/TNFSF10 Protein (TRAIL; Cat# 375-TL-010, R&D Systems) at concentrations of 50 and 100 ng/mL for 18 h with or without pre-treatment with DPP4i (teneligliptin, 3 μM) for 6 h. After 18 h of TRAIL treatment, CCK-8 (#DJDB4000X, Dojindo Molecular Technologies, Tokyo, Japan) solution (10 μL) was added to each well, and the plates were incubated for an additional 1 h. Cell proliferation was calculated as the OD_450_ value of each well as measured using a microculture plate reader (BioTek, Winooski, VT, United States).

**Immunoblot analysis**

To prepare total cell lysates, mouse liver and HepG2 cells were lysed with M-PER reagent (Thermo Fisher Scientific, 78503) and the protein content was measured using the bicinchoninic acid assay kit (Pierce Biotechnology, Rockford, IL, USA). Equal protein amounts from each sample were heat denatured in 5× sample buffer (2% sodium dodecyl sulfate [Tech & Innovation, BSS-9005], 62.5 mM Tris [pH 6.8], 0.01% bromophenol blue, 1.43 mM mercaptoethanol, and 0.1% glycerol), separated on 10% polyacrylamide gels, and electrophoretically transferred onto the polyvinylidene fluoride membranes (Bio-Rad, 1620175). Membrane proteins were extracted using the Mem-PER plus membrane Protein Extraction Kit (Pierce Biotechnology) according to the manufacturer’s instructions. For the liver tissue samples, after blocking, the membranes were incubated with the following antibodies: anti-DPP4 (Abcam, ab129060), and anti- E-cadherin (Santa Cruz, G-10). For HepG2 cells, the membranes were incubated with anti-DPP4 (Thermo Scientific, PA5-42514) and anti-Beta-Actin (ACTB; Sigma-Aldrich, A5441) antibodies. To evaluate the protein level of DR5, whole cell lysates were probed with anti-DR5 (ProSci, 2019) and anti-glyceraldehyde 3-phosphate dehydrogenase (GAPDH; Santa Cruz, sc-32233) antibodies. Immunostaining was performed using the chemiluminescent reagents (SuperSignal West Pico Luminol/Enhancer solution; Thermo Scientific, 34080) and Agfa medical X-ray film (Mortsel, CURIX 60).

**RNA Isolation, cDNA Synthesis, and quantitative real-time polymerase chain reaction**

Total RNA from HepG2 cells and the liver, brown adipose tissue, and perirenal visceral adipose tissue of mice was isolated using TRIzol reagent (Invitrogen, 15596–018), and cDNA synthesis was performed using the High Capacity cDNA Reverse Transcription kit (Applied Biosystems, 4368814) according to the manufacturers’ instructions. The cDNA was then amplified in the ABI 7500 sequence detection system (Applied Biosystems, 4350584) using Power SYBR Green PCR Master Mix (Applied Biosystems, 4367659) with the following cycling conditions: 40 cycles of 95°C for 5 sec, 58°C for 10 sec, and 72°C for 20 sec. Specific primers sequences are listed in the below table. The expression level of genes was normalized to that of β–actin.

**Table.** Forward and reverse sequences designed for qPCR amplification in mouse and human

| **Genes** | **Forward primer (5’ to 3’)** | **Reverse primer (5’ to 3’)** |
| --- | --- | --- |
| Mouse αSMA | CGTGGCTATTCCTTCGTTAC | TGCCAGCAGACTCCATCC |
| Mouse ATF4 | ATGGCGCTCTTCACGAAATC | ACTGGTCGAAGGGGTCATCAA |
| Mouse CHOP | CTGGAAGCCTGGTATGAGGAT | CAGGGTCAAGAGTAGTGAAGGT |
| Mouse colloagen1α1 | CCTGGTAAAGATGGTGCC | CACCAGGTTCACCTTTCGCACC |
| Mouse DR5  (TRAIL-R2) | GTCAGAAGGGAACTGCAAGC | GCATCGACACACCGTATTTG |
| Mouse GAPDH | TGCCTCCTGCACCACCAACT | CCCGTTCAGCTCAGGGATGA |
| Mouse IL-6 | GCCAGAGTCCTTCAGAGAGA | GGTCTTGGTCCTTAGCCACT |
| Mouse MCP-1 | TTAAAAACCTGGATCGGAACCAA | GCATTAGCTTCAGATTTACGGGT |
| Mouse PGC1α | CCGAGAATTCATGGAGCAAT | GTGTGAGGAGGGTCATCGTT |
| Mouse PRDM16 | GCAGATCTGAAGACTTGGG | AAGGAGTAGGCACCTTCTTTCAC |
| Mouse TGF-β | TGACGTCACTGGAGTTGTACGG | GGTTCATGTCATGGATGGTGC |
| Mouse TNF-α | CGTCAGCCGATTTGCTATCT | CGGACTCCGCAAAGTCTAAG |
| Mouse UCP-1 | AGGCTTCCAGTACCATTAGGT | CTGAGTGAGGCAAAGCTGATTT |
| Human ATF4 | ATGACCGAAATGAGCTTCCTG | GCTGGAGAACCCATGAGGT |
| Human β–actin | GGACTTCGAGCAAGAGATGG | AGCACTGTGTTGGCGTACAG |
| Human CHOP | GGAAACAGAGTGGTCATTCCC | CTGCTTGAGCCGTTCATTCTC |
| Human DPP4 | TACAAAAGTGACATGCCTCAGTT | TGTGTAGAGTATAGAGGGGCAGA |
| Human DR5  (TRAIL-R2) | TGCAGCCGTAGTCTTGATTG | GCACCAAGTCTGCAAAGTCA |

**Statistical analysis**

Data are expressed as the mean ± SEM. For animal experiments, data collection and analysis were performed blinded to the experimental conditions. Statistical significance was confirmed using the independent t-test when comparing two groups or the one-way analysis of variance (ANOVA) test when comparing more groups. The degree of relationship between relative mRNA levels of TRAIL-R2, DPP4, biochemical indices, and NAFLD activity score was calculated and presented as Spearman’s rank correlation coefficient (*r*). Multivariate linear regression models were used to determine the independent predictive marker for NAFLD activity score with adjustment for potential confounding factors. *p*-values <0.05 were considered statistically significant. All statistical analyses were conducted using SPSS software, version 23.0, for Windows (IBM Corp., Armonk, NY, USA).

**Supplementary Figures and Figure legends**

**
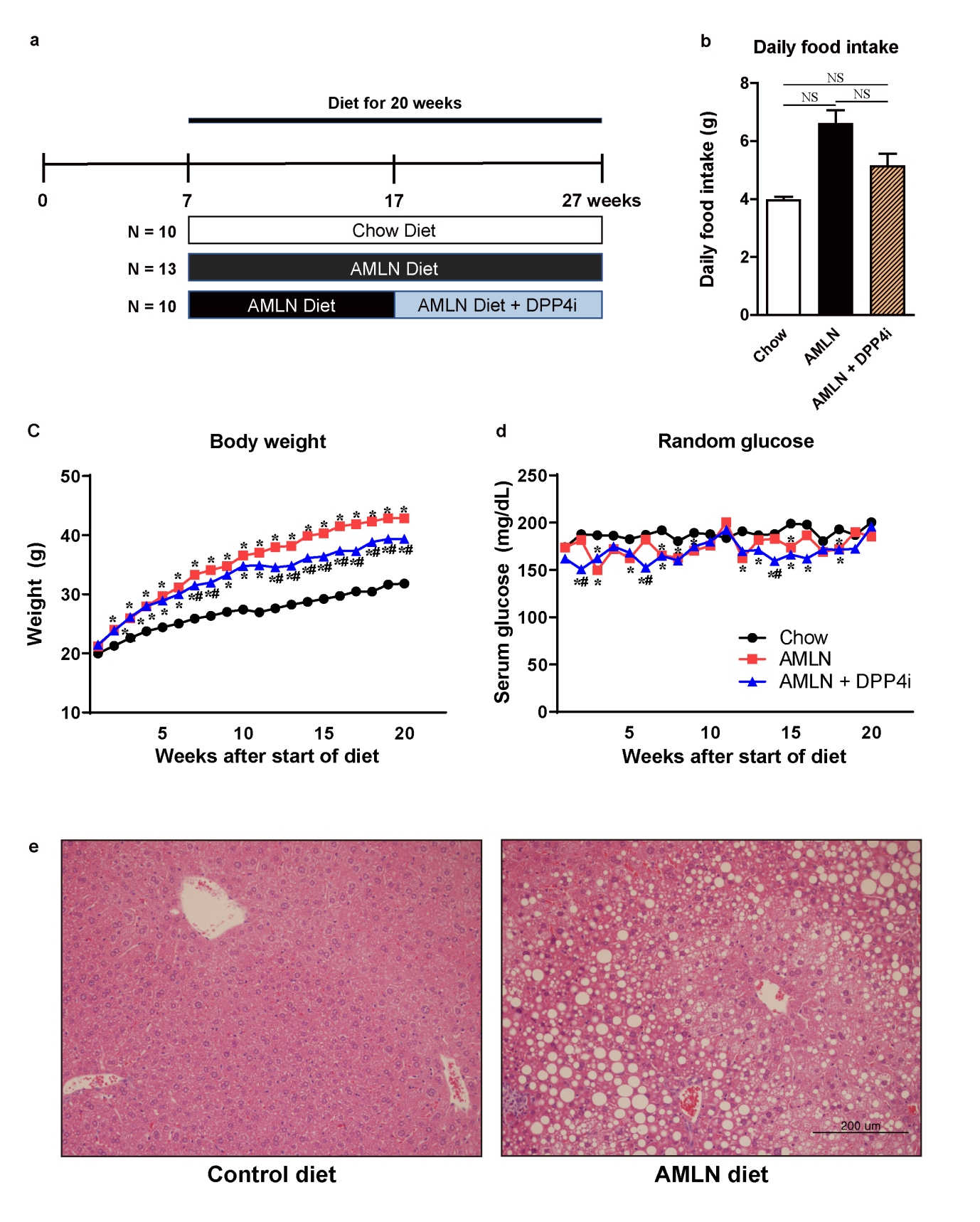
**

**Supplementary Figure S1. Experimental protocol (a) and daily food intake (b), body weight (c), and random glucose levels (d) during experimental course according to diet and DPP4i administration. Representative histopathological liver images by H&E staining (e) in vehicle-treated chow-fed mice and vehicle-treated AMLN-fed mice at 10th weeks of diet administration (200× magnification, scale bar: 200 μm).**

**p* < 0.05 versus vehicle-treated chow-fed mice; #*p* < 0.05 versus vehicle-treated AMLN-fed mice; *NS*, not statistically significant.

Chow, vehicle-treated chow-fed mice; AMLN, vehicle-treated AMLN-fed mice; AMLN + DPP4i, DPP4i-treated (teneligliptin 20 mg/kg of body weight/day by oral gavage for 10 weeks) AMLN-fed mice.


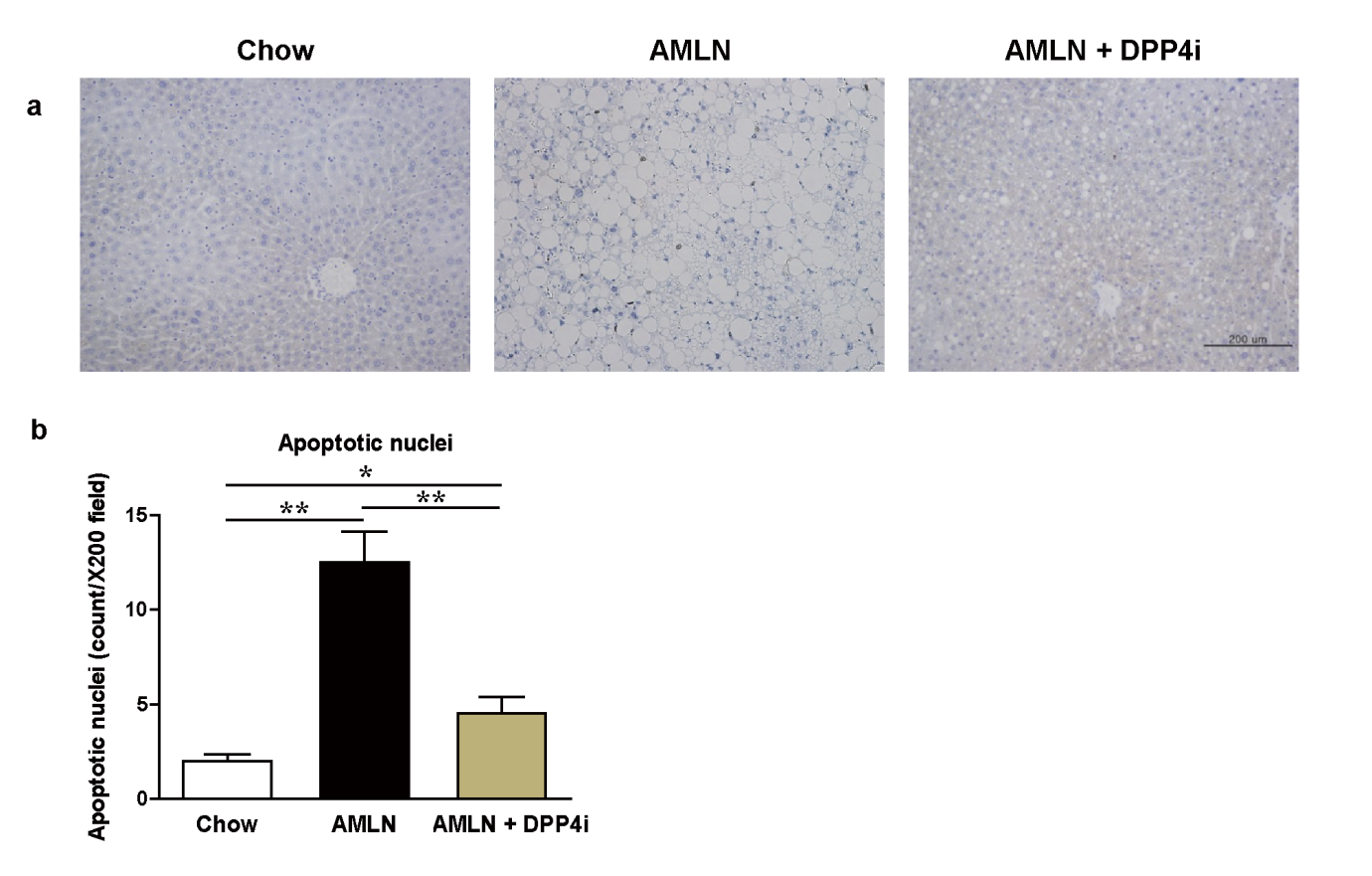


**Supplementary Figure S2.** **DPP4i attenuates liver apoptosis in AMLN diet-induced mouse model of NASH**

Representative liver histopathological images by TUNEL staining (a) of vehicle-treated chow-fed mice, vehicle-treated AMLN-fed mic, and DPP4i-treated AMLN-fed mice (200× magnification, scale bar: 200 μm). The number of TUNEL-positive cells (b) was counted in total 40 random microscopic fields (200×).

Data in a graph are presented as mean ± SEM. **p* < 0.05; ***p* < 0.01.

Chow, vehicle-treated chow-fed mice; AMLN, vehicle-treated AMLN-fed mice; AMLN + DPP4i, DPP4i-treated (teneligliptin 20 mg/kg of body weight/day by oral gavage for 10 weeks) AMLN-fed mice; TUNEL, terminal deoxynucleotidyl transferase dUTP nick-end labeling.


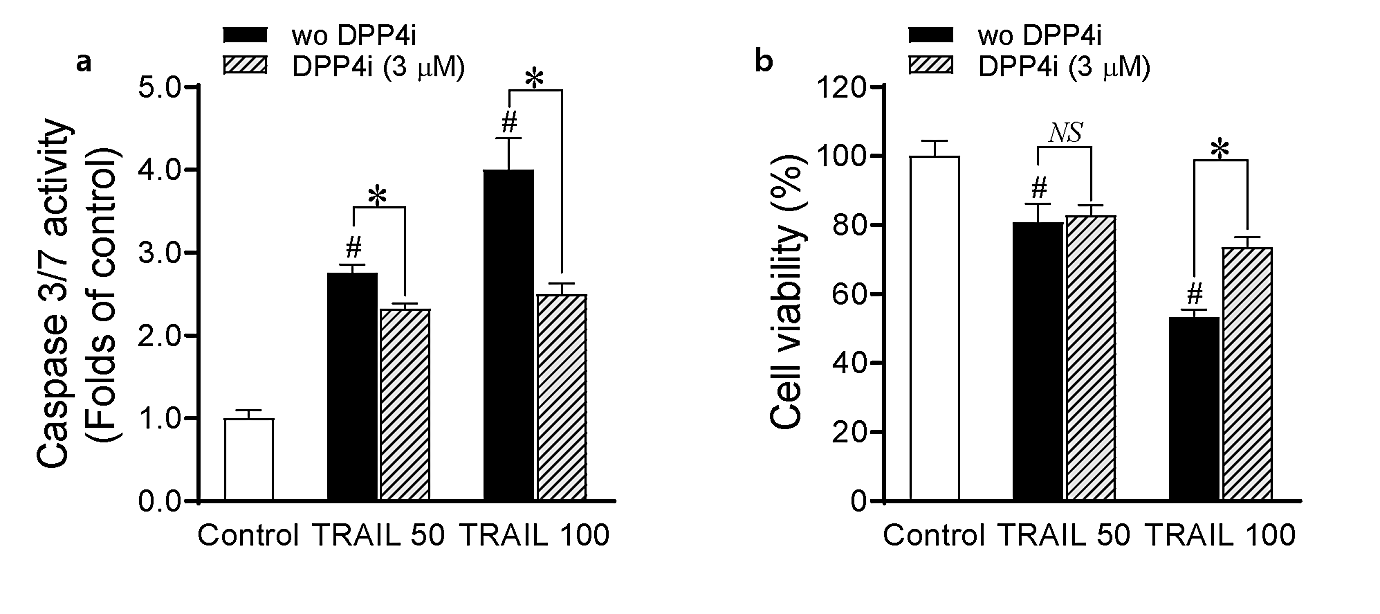


**Supplementary Figure S3. DPP4i treatment attenuates TRAIL-induced apoptosis in hepatocytes**

Graphs showing (a) Caspase 3/7 activity (n=4), and (b) cell viability measured using CCK8 assay (n=4) according to TRAIL and DPP4i treatment.

Data in all graphs are presented as mean ± SEM. ^#^*p* < 0.05 versus control condition; **p* < 0.05 versus TRAIL-treated condition without DPP4i; *NS*, not statistically significant. In all graphs: Control, HepG2 cells, no treatment; TRAIL, HepG2 cells treated with TRAIL-treated (50 and 100 ng/ml) for 18 h; TRAIL + DPP4i, HepG2 cells treated with teneligliptin (3 μM) for 6 h, followed by TRAIL treatment (50 and 100 ng/ml) for 18 h.

DPP4i, dipeptidyl peptidase-4 inhibitors; TRAIL, tumor necrosis factor-related apoptosis-inducing ligand.


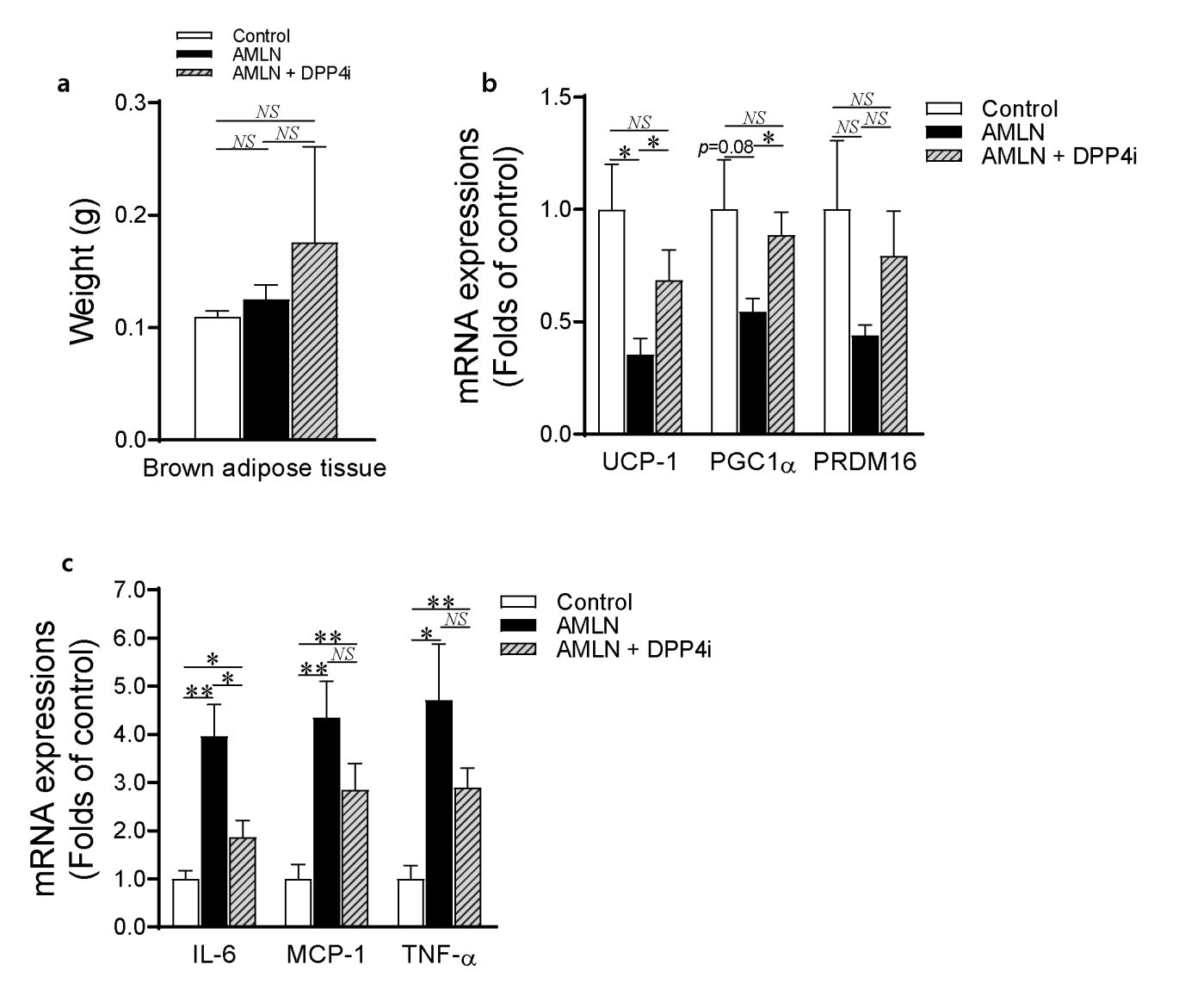


**Supplementary Figure S4. DPP4i treatment is associated with the improvement of brown adipose tissue function and a decrease of visceral adipose tissue inflammation**

Graphs showing (a) weight of brown adipose tissue and relative mRNA expression levels of (b) thermogenic markers: UCP-1, PGC1α, and PRDM16 in brown adipose tissue, and (c) inflammatory markers: IL-6, MCP-1, and TNF-α in perirenal visceral adipose tissue, as determined by qPCR according to the diet type and DPP4i administration.

Data in all graphs are presented as mean ± SEM. **p* < 0.05; ***p* < 0.01; *NS*, not statistically significant.

Chow, vehicle-treated chow-fed mice; AMLN, vehicle-treated AMLN-fed mice; AMLN + DPP4i, DPP4i-treated (teneligliptin 20 mg/kg of body weight/day by oral gavage for 10 weeks) AMLN-fed mice.

DPP4i, dipeptidyl peptidase-4 inhibitors; IL-6, interleukin-6; MCP-1, monocyte chemoattractant protein-1; PGC1α, PPAR-γ coactivator 1-alpha; PRDM16, transcriptional regulator PR domain containing 16; TNF-α, tumor necrosis factor-α; UCP-1, uncoupling protein-1.

**
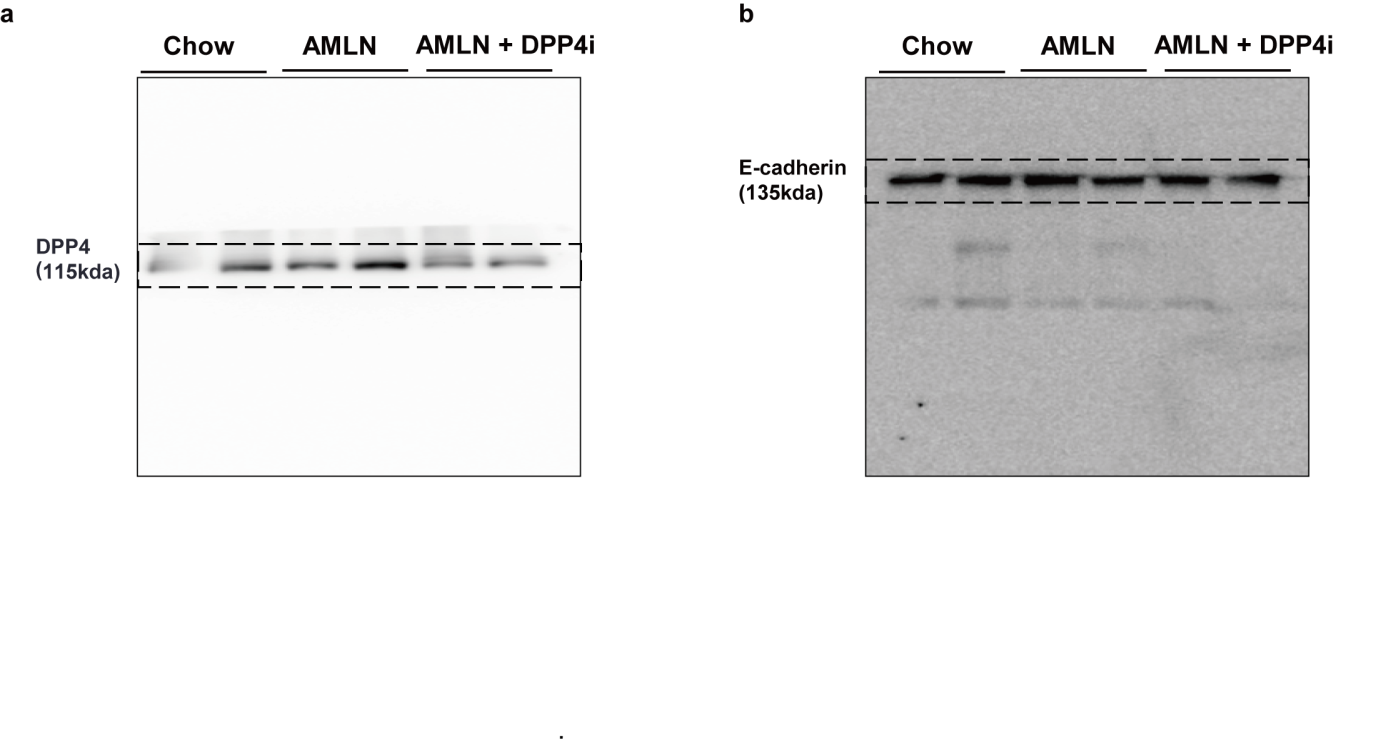
Supplementary Figure S5.** **Raw western blot data corresponding to the cropped blots shown in Figure 2b**

Western blotting of (a) DPP4 from vehicle-treated chow-fed mice, vehicle-treated AMLN-fed mice, and DPP4i-treated AMLN-fed mice; (b) E-cadherin was used as a loading control. The cropped area is shown in inset.

Chow, vehicle-treated chow-fed mice; AMLN, vehicle-treated AMLN-fed mice; AMLN + DPP4i, DPP4i-treated (teneligliptin 20 mg/kg of body weight/day by oral gavage for 10 weeks) AMLN-fed mice.


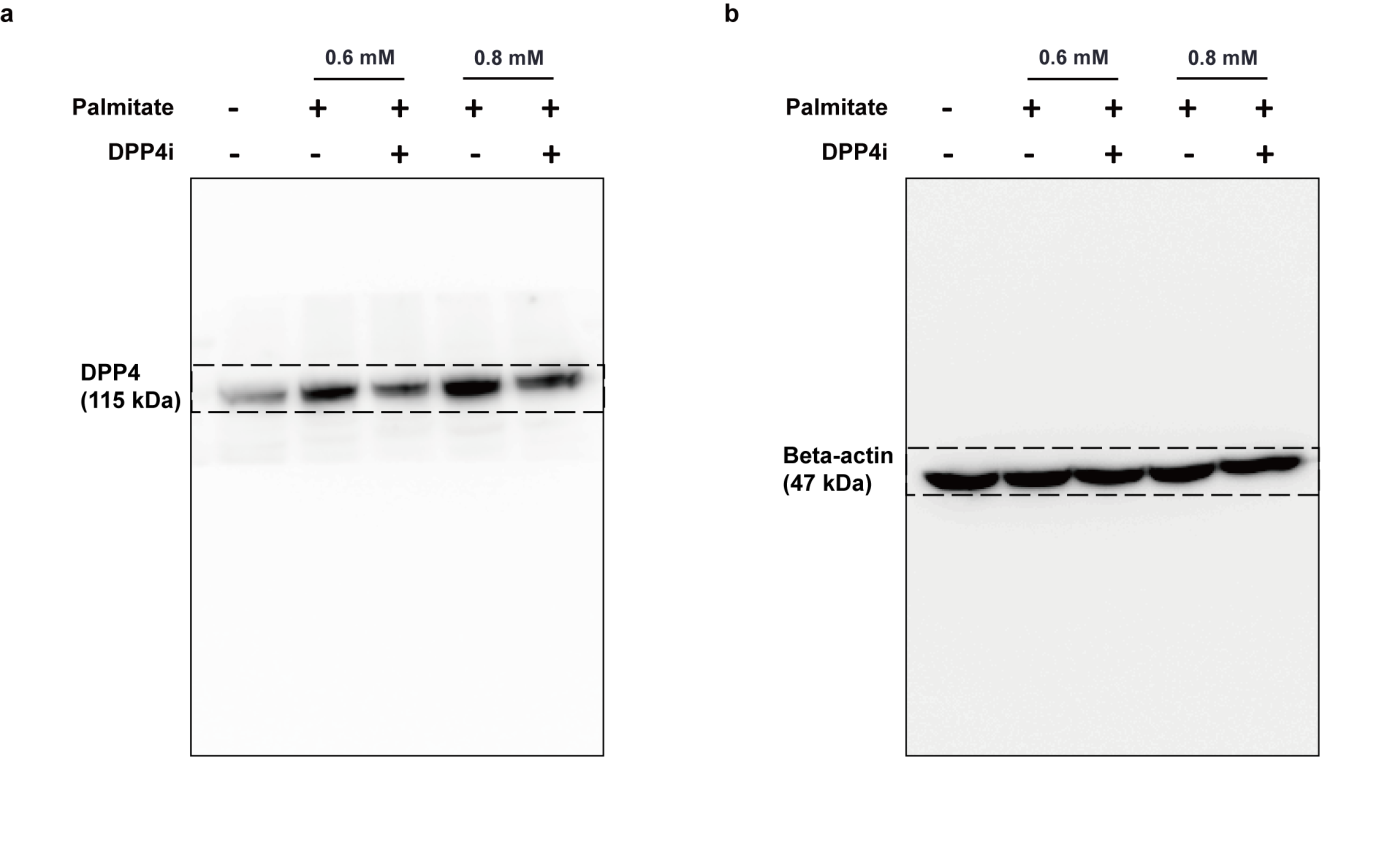


**Supplementary Figure S6.** **Raw western blot data corresponding to the cropped blots shown in Figure 3b**

Western blotting of (a) DPP4 extracted from HepG2 cells treated with palmitate and DPP4i as described in Figure 3b legends; (b) Beta-actin was used as a loading control. The cropped area is shown in inset.

In all graphs: Control, HepG2 cells without specific treatment; Palmitate, 18 h palmitate-treated HepG2 cells; Palmitate + DPP4i, 6 h teneligliptin-treated (3 μM) HepG2 cells followed by 18 h palmitate treatment.


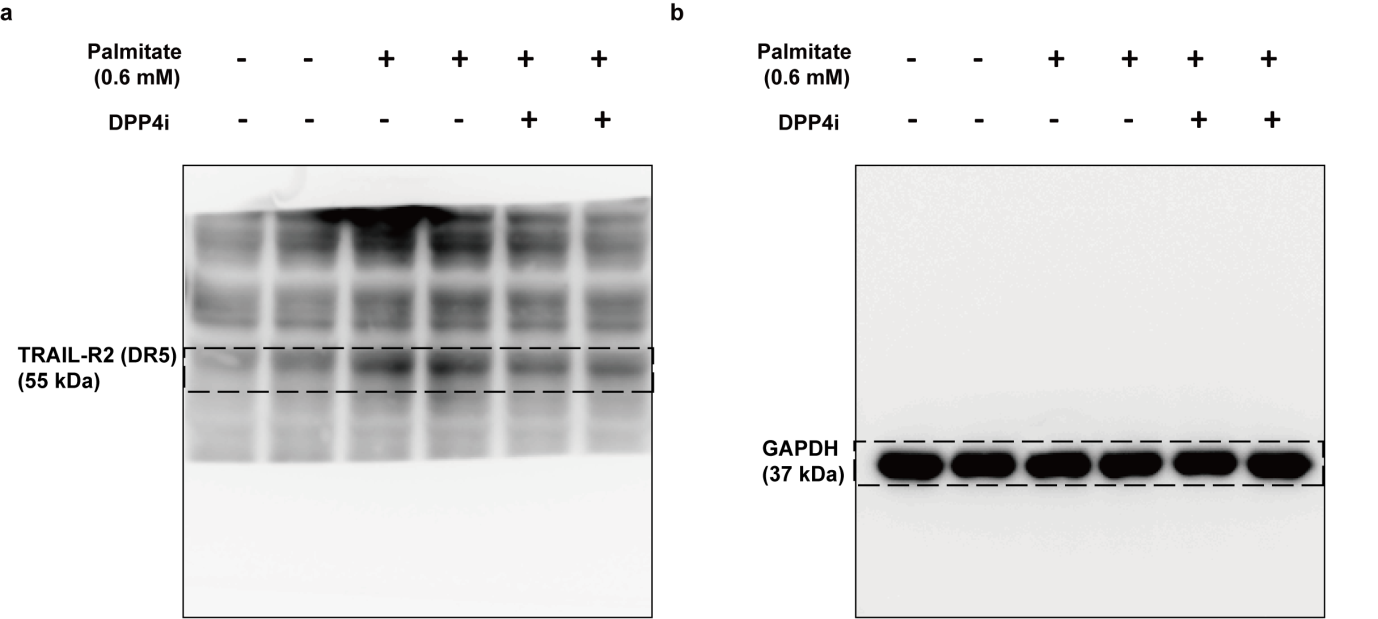


**Supplementary Figure S7.** **Raw western blot data corresponding to the cropped blots shown in Figure 3f**

Western blotting of (a) TRAIL-R2 extracted from HepG2 cells treated with palmitate and DPP4i as described in Figure 3f legends; (b) GAPDH was used as loading control. The cropped area is shown in inset.

In all graphs: Control, HepG2 cells, no treatment; Palmitate, HepG2 cells treated with palmitate (0.6 mM) for 18 h; Palmitate + DPP4i, HepG2 cells treated with teneligliptin (3 μM) for 6 h, followed by palmitate treatment (0.6 mM) for 18 h.
